# Data modeling methods in clinical trials: experiences from the clinical trial methods in neurodegenerative diseases project

**DOI:** 10.1186/1745-6215-12-S1-A15

**Published:** 2011-12-13

**Authors:** Athanasios Anastasiou, Emmanuel Ifeachor, John Zajicek

**Affiliations:** 1 Signal Processing and Multimedia Communications Research Group, University of Plymouth, Drake Circus, Plymouth, UK; 2 Institute of Health Service Research (Peninsula College of Medicine and Dentistry

## Objectives

Clinical trials often generate large and diverse datasets. Data models are used to capture and organise the elements of the data in a meaningful way so that they can be stored and utilised by computer systems and support clinical decision making. This paper presents the data modeling considerations within the ‘Clinical Trial Methods in Neurodegenerative Diseases’ (CTMND) project funded by the NIHR [http://www.ctmnd.org].

The project adopts a holistic approach for the investigation of the suitability and efficiency of clinical observations in neurodegenerative diseases clinical studies. This ongoing research in novel clinical and surrogate outcome measures will be incorporated in an online data collection and analysis system to facilitate clinical trials and relevant research, taking into account, wherever possible, routinely collected NHS data.

This paper presents ongoing research in the project’s data modeling aspects with the following objectives:

1. To review the current state of the art data models for capturing clinical information from the available literature.

2. To compare and contrast their features against the data management requirements of the project and outline the key factors that affected the adoption of a specific model for the CTMND project’s information system.

## Methods

A set of key papers and past reviews were collected from the currently available literature detailing the characteristics of standard data models used in healthcare such as CDISC’s ODM, Health Level 7 and others. The data models and associated approaches were compared and contrasted with each other by taking into account best practices and guidelines emerging from organizations such as the Object Management Group (OMG). Finally, having concluded in a specific modeling approach we were also able to look forward at the possibilities that a particular solution enables and propose a flexible way to model clinical trial data.

## Results

This review highlights a number of key data management and organization considerations that affect the adoption of a specific data model given the project specifications and resource constraints. The key factors were Current Resources, Interoperability (with current and future systems), Documentation and Reference Implementation availability.

## Conclusions

Given the dynamic environment of clinical trials as well as the project’s objectives to propose novel outcome measures, we comment on the suitability of the Dual Model approach for the efficient organization of clinical study data but more importantly for its flexibility in modeling novel outcomes with minimal software maintenance. According to this approach, a handful of elementary data structures (numbers, character sequences, lists, trees and others) are made available to a higher level model that is responsible for their ordering and semantics. Additionally we provide specific details regarding system implementation.

